# In Vitro Modulation of Autophagy by New Antioxidant Nitrones as a Potential Therapeutic Approach for the Treatment of Ischemic Stroke

**DOI:** 10.3390/antiox13080946

**Published:** 2024-08-03

**Authors:** Sara Izquierdo-Bermejo, Beatriz Chamorro, María Dolores Martín-de-Saavedra, Miguel Lobete, Francisco López-Muñoz, José Marco-Contelles, María Jesús Oset-Gasque

**Affiliations:** 1 Department of Biochemistry and Molecular Biology, Faculty of Pharmacy, Complutense University of Madrid, Plaza Ramón y Cajal s/n, Ciudad Universitaria, 28040 Madrid, Spain; sizqui03@ucm.es (S.I.-B.); beatrcha@ucm.es (B.C.); marmar68@ucm.es (M.D.M.-d.-S.); milobete@ucm.es (M.L.); 2 Instituto de Investigación Sanitaria del Hospital Clínico San Carlos, 28040 Madrid, Spain; 3 Faculty of Health Sciences—HM Hospitals, Camilo José Cela University, Villafranca del Castillo, 28692 Madrid, Spain; flopez@ucjc.edu; 4 Instituto Universitario de Investigación en Neuroquímica, Complutense University of Madrid, Ciudad Universitaria, 28040 Madrid, Spain; 5 HM Hospitals Health Research Institute, 28015 Madrid, Spain; 6 Neuropsychopharmacology Unit, “Hospital 12 de Octubre” Research Institute, 28041 Madrid, Spain; 7 Laboratory of Medicinal Chemistry, Institute of Organic Chemistry (CSIC), C/Juan de la Cierva 3, 28006 Madrid, Spain; jlmarco@iqog.csic.es; 8 Center for Biomedical Network Research on Rare Diseases (CIBERER), Carlos III Health Institute (ISCIII), 28029 Madrid, Spain

**Keywords:** autophagy, cerebral ischemia, hypoxia, neuroprotection, nitrones, stroke, therapeutic agents

## Abstract

Stroke is a leading cause of death worldwide, yet current therapeutic strategies remain limited. Among the neuropathological events underlying this disease are multiple cell death signaling cascades, including autophagy. Recent interest has focused on developing agents that target molecules involved in autophagy to modulate this process under pathological conditions. This study aimed to analyze the role of autophagy in cell death induced by an in vitro ischemia–reperfusion (IR) model and to determine whether nitrones, known for their neuroprotective and antioxidant effects, could modulate this process. We focused on key proteins involved in different phases of autophagy: HIF-1α, BNIP3, and BECN1 for induction and nucleation, LC3 for elongation, and p62 for degradation. Our findings confirmed that the IR model promotes autophagy, initially via HIF-1α activation. Additionally, the neuroprotective effect of three of the selected synthetic nitrones (quinolylnitrones **QN6** and **QN23**, and homo-bis-nitrone **HBN6**) partially derives from their antiautophagic properties, demonstrated by a downregulation of the expression of molecular markers involved in various phases of autophagy. In contrast, the neuroprotective power of cholesteronitrone **ChN2** seems to derive from its promoting effects on the initial phases of autophagy, which could potentially help inhibit other forms of cell death. These results underscore the importance of autophagy modulation in neuroprotection, highlighting the potential of inhibiting prodeath autophagy and promoting prosurvival autophagy as promising therapeutic approaches in treating ischemic stroke clinically.

## 1. Introduction

Stroke is a cerebrovascular disease caused by the disruption of blood flow in one or more brain regions [1,2]. It is currently the second leading cause of death and the third leading cause of disability worldwide [3].

The most common type of stroke is ischemic stroke [4,5], characterized by a series of biochemical processes resulting from vessel occlusion collectively referred to as the ischemic cascade [4,6]. Due to the lack of glucose and oxygen, brain cells undergo energy deficits and alterations in ionic and acid–base balance [7,8]. This leads to glutamate excitotoxicity [7,8,9], oxidative and nitrosative stress [7,8], neuroinflammation [4,9], and blood–brain barrier (BBB) dysfunction [4,8].

These neuropathological events trigger various types of cell death, including necrosis, apoptosis, autophagy, pyroptosis, and ferroptosis [8,10]. The role of autophagy is particularly interesting due to its dual nature [11]. At appropriate levels, it is essential for maintaining homeostasis, degrading nonfunctional proteins and organelles, and responding to cellular stress [8,12,13]. However, excessive activation of autophagy can induce cell death [6,13]. Thus, while autophagy may contribute to cell death resulting from ischemia, it also promotes survival during the early stages of the ischemic cascade and functional recovery post-stroke (e.g., by reducing neurite degeneration and blocking mechanisms of necrosis and apoptosis) [1,9,11].

The process of autophagy is triggered by situations such as fasting, energy depletion, or other cellular stress factors (e.g., hypoxia) [1,6,11] and occurs in five stages: (1) initiation or induction, (2) nucleation, (3) expansion and elongation of the autophagosome membrane, (4) maturation (closure and fusion with the lysosome), and (5) degradation of intravesicular contents [6,14]. Each of these stages presents specific molecular targets for the modulation of autophagy with significant therapeutic potential (Figure S1) [14].

Hypoxia-inducible factor 1 (HIF-1) is a heteromeric transcription factor that regulates gene expression in response to hypoxia [15]. It consists of a constitutively expressed HIF-1β subunit and an inducibly expressed HIF-1α subunit [15]. Under hypoxic conditions, HIF-1α ubiquitination is inhibited, allowing it to accumulate, dimerize with HIF-1β, and activate the transcription of a broad range of hypoxia-responsive genes [16]. Recent studies indicate that HIF-1α promotes programmed cell death during cerebral ischemia by inducing Bcl-2 family members like BCL2 Interacting Protein 3 (BNIP3), which regulates autophagy nucleation [17,18,19,20,21,22]. Autophagy can also be triggered by inhibiting mTOR complex 1 (mTORC1) [23], leading to activation of the UNC-51-like kinase (ULK1) complex (Atg1) [24] or class III phosphoinositide 3-kinase (PI3KIII) association with Beclin-1 (Becn1 or Atg6) and Atg14 [10,11]. Under normal physiological conditions, Becn1 is inhibited by the antiautophagic regulator Bcl-2 [25,26]. However, during hypoxic stress, BNIP3 binds to Bcl-2, disrupting the Bcl-2/Becn1 interaction and facilitating autophagosome formation [20] (Figure S1). Consequently, Becn1 and BNIP3 are widely utilized as markers for the induction and nucleation phases of autophagy. Preautophagosome elongation is then driven by two ubiquitination cascades, resulting in the formation of the autophagosomes, which engulf the cargo to be degraded [25]. During this process, the cytosolic isoform of Microtubule-Associated Protein 1 Light Chain 3 (LC3), known as LC3-I, conjugates with phosphatidylethanolamine (PE) molecules in a reaction mediated by Atg3 and Atg7 [6,11] (Figure S1). The resulting lipidated isoform, LC3-II, incorporates into the autophagosome membrane [12]. Therefore, the LC3-II/LC3-I ratio is the most commonly used indicator for studying autophagic flux [6]. In turn, LC3 acts as a receptor for p62, a cytosolic protein that recruits ligands destined for degradation via autophagy, thereby mediating this process in which p62 itself is also degraded [6,9,27] (Figure S1). Thus, p62 levels directly correlate with the rate of autolysosomal degradation.

Current therapeutic strategies for ischemic stroke focus on the recanalization of occluded blood vessels and reperfusion of the affected brain tissue, either through pharmacological dissolution (thrombolysis) or mechanical removal (thrombectomy) of thrombi [28]. However, these approaches have many limitations. For instance, recombinant tissue plasminogen activator (rtPA) poses hemorrhagic effects at the interface between blood flow and the brain (thereby increasing BBB permeability) [29] and neurotoxicity by potentiating NMDA receptor signaling, leading to cell death [30]. Moreover, recanalization strategies exacerbate the negative consequences of restoring blood flow, such as oxidative stress [10,31]. In contrast, neuroprotective strategies aim to mitigate pathogenic mechanisms at various stages of the ischemic cascade to prevent cell death by reducing the activation of metabolic pathways that contribute to cellular damage (e.g., excitotoxicity, neuroinflammation, oxidative stress) and enhancing endogenous cytoprotective responses (e.g., synthesis of growth factors, stimulation of neurogenesis and angiogenesis) [1,8,32]. Ideally, combining reperfusion therapies with neuroprotective agents could extend the therapeutic window of rtPA, thereby prolonging brain tissue viability and reducing potential side effects [7,33].

**Figure 1:**
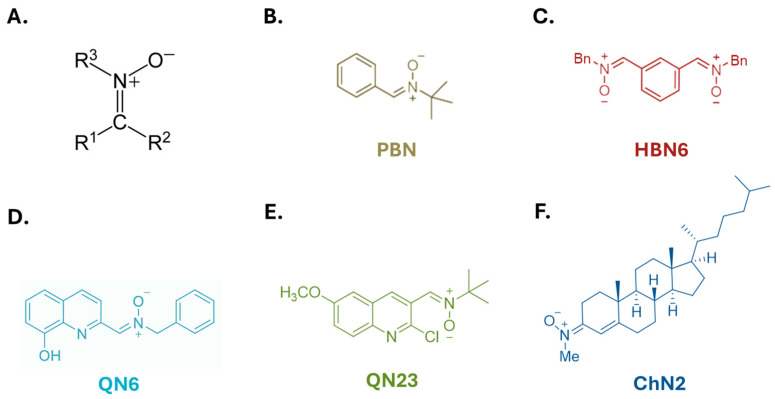
Structures of (**A**) the nitrone group, (**B**) α-phenyl-N-tert-butylnitrone (**PBN**), (**C**) homo-bis-nitrone 6 (**HBN6**), (**D**) quinolylnitrone **QN6**, (**E**) quinolylnitrone **QN23,** and (**F**) cholesteronitrone **ChN2**.

Among these neuroprotective agents are nitrones, which are imine N-oxides (see Figure 1) capable of scavenging free radicals and reactive oxygen species (ROS) [34]. Nitrone **NXY-059**, derived from α-phenyl-N-tert-butylnitrone (**PBN**), reached phase III clinical trials but was discarded due to a lack of significant improvements in patients [35]. Challenges in translating the neuroprotective potential of these compounds from laboratory experiments to clinical settings include limitations in preclinical experimental models [2,36] and complexities in designing clinical trials for stroke treatment [31,37,38].

Our research group has focused on the synthesis and pharmacological characterization of new nitrones for the treatment of ischemic stroke [34,39,40,41,42,43,44]. Recently, we studied the neuroprotective effects of bis-nitrones [34] and tris-nitrones [40] derived from **PBN**, cholesteronitrones [38,41,44], and quinolylnitrones [39,42,43,44], among others.

In this study, our objectives were threefold: (1) to investigate the potential involvement of autophagy in cell survival or death in an in vitro model of cerebral ischemia–reperfusion by analyzing key markers across different autophagic stages; (2) to identify the autophagy inducer in our model, specifically exploring HIF-1αs’ role; and (3) to analyze the potential modulatory effects on autophagy of four highly active nitrones (**ChN2**, **QN6**, **QN23**, and **HBN6**) from distinct structural groups (see Figure 1) and their implication in the overall neuroprotective effects of these compounds. These nitrones have previously demonstrated efficacy against necrosis, apoptosis, and reactive oxygen species (ROS) production in in vitro models [34,38,39,41,43,44] and significant neuroprotective potential in in vivo ischemia models [34,39,41,42,45]. Additionally, we compared their potential effects on autophagy with those of two reference compounds: **PBN** (the parent nitrone providing their structural basis) and N-acetylcysteine (**NAC**), a well-known antioxidant [46].

## 2. Materials and Methods

### 2.1. Neuroblastoma Cell Cultures

SH-SY5Y human neuroblastoma cells (ATCC CRL-2266) (LGC Limited, Luckenwalde, Germany) were cultured in flasks containing Dulbecco’s/Ham’s F12 (Gibco, Life Technologies, Madrid, Spain), supplemented with GlutaMAX 2.5 mM (Gibco, Life Technologies, Madrid, Spain), 1% antibiotic–antimycotic solution (100 mg/mL streptomycin, 100 µL/mL penicillin, and 0.25 mg amphotericin B) (Gibco, Life Technologies, Madrid, Spain), 1% gentamicin at 15 mg/mL (Sigma-Aldrich, Madrid, Spain), and 10% fetal bovine serum (FBS) (Gibco, Life Technologies, Madrid, Spain) following established protocols [34,44]. Cultures were maintained at 37 °C in a humidified atmosphere of 5% CO_2_ and 95% air. The culture medium was changed every 2 days and, upon reaching confluence, cells were subcultured into new flasks after treatment with 0.25% trypsin-EDTA (Gibco, Life Technologies, Madrid, Spain). For each experiment, SH-SY5Y cells were seeded into either 6- or 96-well plates at densities of 1.5 × 10^6^ cells/well or 0.5 × 10^5^ cells/well, respectively, depending on the assay.

### 2.2. Neuroblastoma Cell Cultures’ Exposure to Oxygen–Glucose Deprivation (OGD)

To induce experimental ischemia (I), SH-SY5Y cells underwent OGD for 3 h. This was accomplished by using glucose-free Dulbecco’s medium (Gibco, Life Technologies, Madrid, Spain) and placing the plates in an anaerobic chamber with a gas mixture of 95% N_2_/5% CO_2_, maintained at 37 °C and 0.15 bar pressure [43,44]. Following the OGD period, glucose-free medium was replaced by the standard oxygenated medium and the compounds were added to the wells at different concentrations. Cells were kept under normoxic conditions to simulate reperfusion for 3, 6, or 24 h, depending on the experiment. It is noteworthy that while the term “ischemia–reperfusion” (IR) is utilized herein, a more precise term for cellular models would be “oxygen and glucose resupply” (OGD-R). Controls were performed by preserving SH-SY5Y cells in glucose-containing Dulbecco’s medium under normoxic conditions for 3 h, followed by a medium change and further incubation for 3, 6, or 24 h. Additionally, the same concentrations of the vehicles used for compound dissolution (ethanol for **ChN2** and dimethyl sulfoxide for the rest of the molecules) were included in all experiments (final concentration < 1%).

### 2.3. Evaluation of Cell Viability

To assess the involvement of autophagy in cell survival under basal conditions and in response to the ischemia–reperfusion (IR) model, as well as the influence of autophagy modulation exerted by the compounds, cell viability was measured in SH-SY5Y human neuroblastoma cells seeded in 96-well culture plates at a density of 0.5 × 10^5^ cells/well. Following exposure to either OGD-R or a control treatment, **ChN2**, **QN6**, **QN23**, **HBN6**, **PBN**, and **NAC** were added at concentrations ranging from 0.1 to 100 µM. Subsequently, cell viability was evaluated using the Cell Proliferation Kit II (XTT) (ThermoFisher, Madrid, Spain), which quantifies cellular respiration by measuring the ability of metabolically active cells to reduce a yellow tetrazolium salt to an orange formazan dye [40]. Following a 2 h incubation period with the XTT solution (0.3 mg/mL) at 37 °C with 5% CO_2_ and 95% air (*v*/*v*), absorbances at 450 nm and 650 nm (used as references) were measured using a spectrophotometer (Power-Wave XS microplate-reader, BioTek Instruments, Madrid, Spain) [45,46]. A viability baseline of 100% was set by the control normoxic cells treated solely with standard Dulbecco’s medium.

### 2.4. Western Blotting

To explore autophagy-induced cell death and the modulatory effect of the tested compounds, Western blotting was employed. SH-SY5Y cells were seeded in 6-well plates at a density of 1.5 × 10^6^ cells/well. Following exposure to OGD-R conditions, and the subsequent addition of the compounds at different concentrations, cellular extracts were prepared using RIPA lysis buffer (50 mM Tris, 150 mM NaCl, 0.5% sodium deoxycholate, 0.1% SDS, 50 mM NaF, 100 μM NaVO_3_, 500 μM PMSF, 5 μg/L aprotinin, 0.025 μg/L leupeptin, and 10% Triton). After scraping the wells, protein concentration was determined using the Bradford method [47], and sample volumes were adjusted to ensure uniform gel loading. Subsequently, Laemmli 2× buffer and β-mercaptoethanol were added to the samples, which were then heated at 95 °C for 5 min, and loaded onto 8%, 10%, and 12% polyacrylamide gels. Following electrophoretic separation, proteins were transferred to polyvinylidene fluoride (PVDF) membranes activated in methanol and blocked with TBS-Tween (19 mM Tris, 137 mM NaCl, 2.7 mM KCl, 0.1% Tween20) and 3% bovine serum albumin (BSA) for 1 h. Finally, the membranes were incubated with primary antibodies overnight at 4 °C, followed by incubation with secondary antibodies for 1 h at room temperature. Immunoreactive bands were visualized by enhanced chemiluminescence (ECL) using SuperSignal West Pico PLUS (Bio-Rad, Madrid, Spain), a chemiluminescent horseradish peroxidase substrate [48]. Luminescence signals were captured with a gel analysis system (VWR^®^ Imager CHEMI Premium, Madrid, Spain) and quantified using ImageJ (Fiji) 1.50b software (NIH, Stapleton, NY, USA). Details regarding the antibodies used in these experiments, including their dilutions, are provided in Table 1.

### 2.5. Statistical Analyses

The data obtained from cell cultures are expressed as the means ± SEMs of the results derived from a minimum of three independent experiments with different cell batches, each one conducted in duplicate or triplicate. Statistical comparisons among the various experimental groups were performed using a one-way analysis of variance (ANOVA), followed by a Holm–Sidak post hoc test, utilizing GraphPad Prism version 8.0 (GraphPad Software, San Diego, CA, USA). A significance threshold of *p* < 0.05 was employed. Additionally, regression analyses and statistics were carried out using the Pearson correlation test, also within GraphPad Prism version 8.0 (GraphPad Software, San Diego, CA, USA).

## 3. Results

### 3.1. Role of Autophagy in an In Vitro Model of Ischemia and Ischemia–Reperfusion Injury

To investigate the involvement of autophagy in the molecular mechanisms underlying the ischemia–reperfusion (IR) experimental model, we analyzed the expression of proteins associated with three distinct phases of autophagy in SH-SY5Y cells across various experimental conditions using Western blot. Specifically, we used BNIP3 and Becn1 as markers for the initiation and nucleation phases, LC3 for the elongation phase, and p62 for the degradation phase. To validate our results, we employed two autophagic modulators: (1) chloroquine (CQ) at 20 μM, which inhibits autophagic flux by neutralizing the lysosomal pH [49] and preventing autophagosome–lysosome fusion [50], and (2) rapamycin (Rapa) at 40 nM, used as a positive control for autophagy due to its mTOR inhibitory effect [1,6,51].

**Figure 2:**
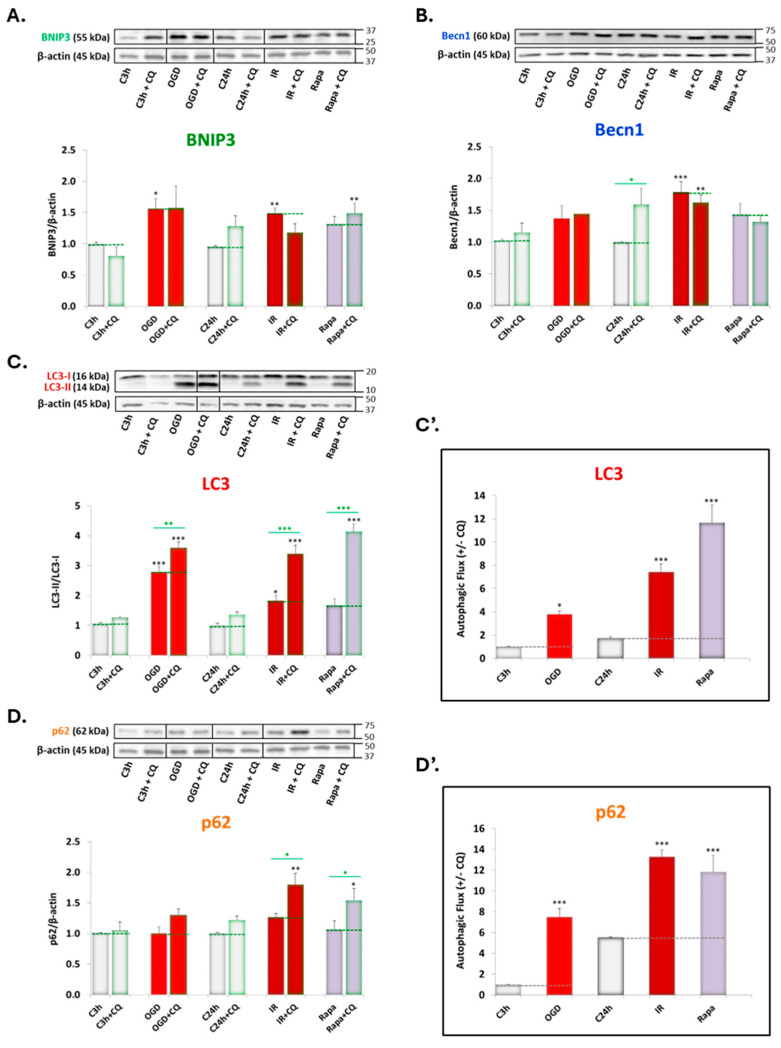
Effect of ischemia (OGD), reperfusion (IR), and treatment with 20 μM chloroquine (CQ) and 40 nM rapamycin (Rapa) on the expression of (**A**) BNIP3, (**B**) Becn1, (**C**) LC3, and (**D**) p62 in SH-SY5Y cells. BNIP3, Becn1, and p62 (panels (**A**,**B**,**D**)) were normalized to β-actin expression. LC3 lipidation is expressed as the LC3-II/LC3-I ratio (panel (**C**)). Autophagic flux of LC3 (panel (**C’**)) and p62 (panel (**D’**)) is the difference in protein expression with and without 20 μM CQ. Values shown are the means ± SEMs of 4 experiments performed in duplicate. Black asterisks (*): significant differences in protein expression among experimental conditions relative to controls (C3h and C24h). Green asterisks (*): significant differences in protein expression under specific experimental conditions compared to cotreatment with CQ (* *p* < 0.05; ** *p* < 0.01; *** *p* < 0.001; one-way ANOVA followed by Holm–Sidak post hoc test).

As shown in Figure 2, exposure to 3 h OGD and 24 h reperfusion (IR) significantly upregulated BNIP3 expression (Figure 2A) compared to the control conditions. Similarly, Becn1 also exhibited a statistically significant increase under IR conditions (Figure 2B). Treatment with rapamycin tended to enhance the expression of both molecular targets, although its effect was not statistically significant (Figure 2A,B).

Additionally, exposure to OGD conditions significantly increased LC3 lipidation, which partially reverted upon reperfusion (Figure 2C). Furthermore, treatment with CQ significantly enhanced the accumulation of LC3-II under both OGD and IR conditions, as well as combined treatment with Rapa (Figure 2C).

Finally, we observed a significant increase in p62 expression under IR conditions and with rapamycin treatment when combined with CQ (Figure 2D).

Given that CQ treatment only increased the expression of LC3 and p62, we calculated the autophagic flux by subtracting the expression levels of these proteins in the presence and the absence of 20 μM CQ. The results indicated a highly significant increase in autophagic flux for both LC3 (Figure 2C’) and p62 (Figure 2D’) under OGD conditions and, mainly, under IR conditions and rapamycin treatment compared to controls.

**Figure 3:**
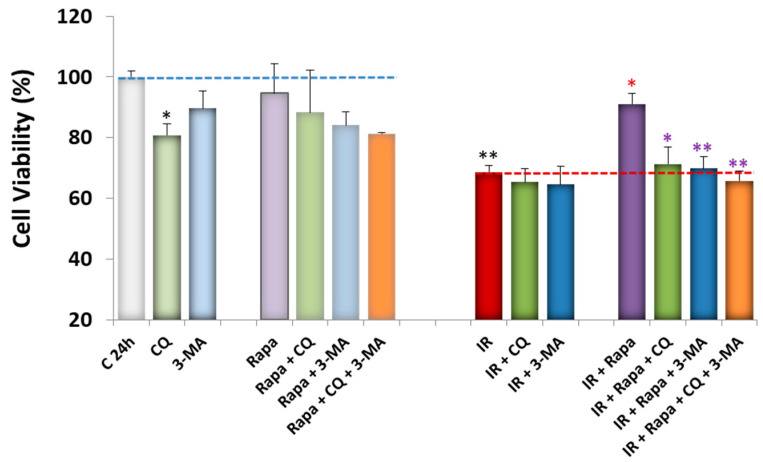
Effect of various autophagy modulators (CQ 20 μM, Rapa 40 nM, and 3-MA 5 mM) on the viability of SH-SY5Y cells under basal conditions (**left**) and IR model conditions (**right**). Values shown are the means ± SEMs of four experiments performed in triplicate. Black asterisks (*): significant differences in cell viability compared to the control condition (C24h). Red asterisks (*): significant differences in cell viability compared to IR. Purple asterisks (*): significant differences in cell viability compared to Rapa treatment alone under IR conditions. (* *p* < 0.05; ** *p* < 0.01; one-way ANOVA followed by Holm–Sidak post hoc test).

In light of the observed increase in autophagic flux, we sought to determine how this related to cell viability under various experimental conditions (Figure 3).

Under basal conditions, CQ significantly reduced metabolic capacity, highlighting the positive effects of autophagy on cell survival in the absence of damage (Figure 3). This effect was also observed, though to a lesser extent, in the presence of 5 mM 3-methyladenine (3-MA), an inhibitor of class III PI3K involved in the initiation phase of autophagy [52] (Figure 3).

As expected, exposure to IR conditions significantly decreased cell viability as well (Figure 3). CQ and 3-MA slightly reduced cell survival in the IR model, but these reductions were not statistically significant (Figure 3).

Rapamycin treatment, which had no observable effects under basal conditions, significantly reversed the IR-induced decrease in viability (Figure 3). Interestingly, this increase in cell metabolic capacity induced by rapamycin was reversed by treatment with CQ and 3-MA, as well as by their combination (Figure 3).

### 3.2. Potential Role of HIF-1α Stabilization in Triggering Ischemia-Induced Autophagy

**Figure 4:**
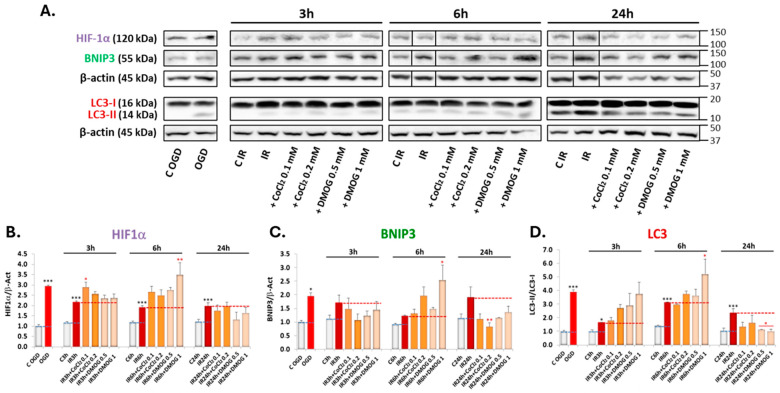
Effect of prolyl hydroxylase inhibitors CoCl_2_ and DMOG on the expression of (**A**,**B**) HIF-1α, (**A**,**C**) BNIP3, and (**A**,**D**) LC3 in SH-SY5Y cells after exposure to IR with different reperfusion times (3, 6, and 24 h). Values shown are the means ± SEMs of three experiments performed in duplicate. Black asterisks (*): significant differences in protein expression between experimental conditions and their respective controls. Red asterisks (*): significant differences in protein expression between experimental conditions and their IR control. (* *p* < 0.05; ** *p* < 0.01; *** *p* < 0.001; one-way ANOVA followed by Holm–Sidak post hoc test).

After confirming that the IR model could activate autophagy, we investigated whether HIF-1α, a key transcription factor in the cellular response to hypoxia [53], was involved in this process. We aimed to test the hypothesis that post-ischemic HIF-1α upregulation was responsible for inducing autophagy under OGD and IR conditions. To test this, we analyzed HIF-1α protein expression by Western blot after subjecting cells to 3 h of OGD followed by various reperfusion times (3, 6, and 24 h) (Figure 4).

Under normoxic conditions, HIF-1α is hydroxylated by oxygen-dependent prolyl hydroxylases (PHDs), leading to its degradation and clearance from the cell [54,55]. In hypoxic conditions, the activity of PHDs is reduced, resulting in the accumulation of HIF-1α [55,56]. Thus, to better visualize the potential increase in HIF-1α levels under ischemic conditions, we conducted this study with and without two well-known PHD inhibitors (PHDIs): cobalt chloride (CoCl_2_) at 0.1 and 0.2 mM, and dimethyoxalylglycine (DMOG) at 0.5 and 1 mM [57].

As shown in Figure 4B, exposure to OGD significantly upregulated HIF-1α expression, which progressively decreased as reperfusion time increased from 3 to 24 h. Accordingly, PHDIs did not exhibit any significant effect on HIF-1α protein levels after 24 h of reperfusion, with the most notable increase induced by DMOG at 6 h of IR (Figure 4B). These effects are similar to those observed by Ayrapetov et al. (2011) in their study on radioprotection [57].

The effect of PHDIs on BNIP3 expression mirrored their impact on HIF-1α expression at 6 h of IR (Figure 4C). However, this pattern was not observed at other time points. At both 3 and 24 h of IR, BNIP3 expression decreased in the presence of PHDIs, with a significant reduction noted at 24 h (Figure 4C).

Finally, both CoCl_2_ and DMOG increased LC3 lipidation at 3 h of IR (Figure 4D). At 6 h, only DMOG at 1 mM significantly increased LC3-II accumulation, while at 24 h, LC3 lipidation decreased in the presence of both PHDIs (Figure 4D).

These findings suggest that HIF-1α stabilization is crucial for triggering autophagy under IR conditions during early reperfusion, but at longer reperfusion times, IR-induced autophagy becomes independent on HIF-1α and may be maintained by other mechanisms, such as the activation of AMPK and the inhibition of mTOR phosphorylation [13,58].

### 3.3. Effects of Nitrones on Ischemia–Reperfusion-Induced Autophagy and Relation to Cell Viability

#### 3.3.1. Effects on BNIP3, Becn1, LC3, and p62 Expression and Autophagic Flux

After confirming the involvement of autophagy in the in vitro IR model, we investigated the impact of the nitrones studied in this work on modulating different phases of IR-induced autophagy. We evaluated the effects of these compounds at concentrations of 1 and 10 μM (based on EC_50_ values from previous studies [34,43,44]) on the expression of key autophagy-related proteins, chosen as markers of the induction, nucleation, elongation, and degradation phases. Furthermore, we conducted combined treatments with 20 μM CQ to assess whether the compounds influenced autophagic flux.

Firstly, none of the tested nitrones showed significant modulatory effects on the expression of induction/nucleation markers (Figure S2). None of the compounds altered BNIP3 expression at 1 or 10 μM, except for **QN6**, which significantly decreased it at 10 μM (Figure S2B). Regarding Becn1, all compounds at 1 μM exhibited a trend towards reduced protein expression, with significant effects observed only for **NAC** and **QN6** (Figure S2C). However, this inhibitory effect was not observed at 10 μM for any of the tested compounds (Figure S2D). Treatment with CQ did not induce significant changes in BNIP3 or Becn1 expression for any of the compounds either (Figure S2).

**Figure 5:**
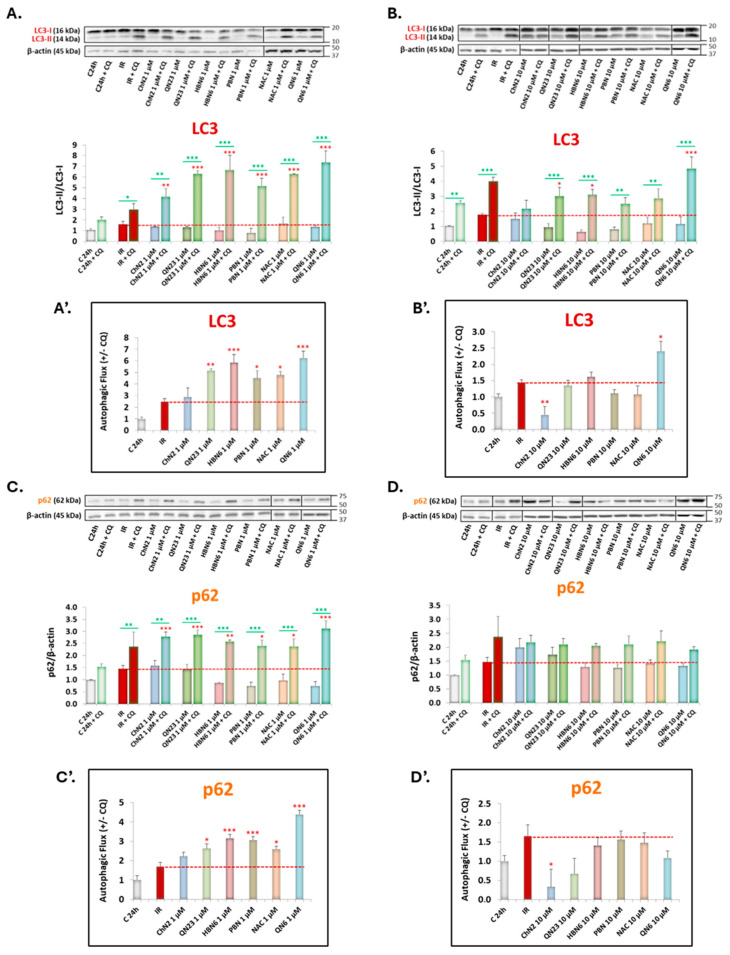
Effect of the assessed compounds at 1 and 10 μM on (**A**,**B**) LC3 lipidation and (**C**,**D**) p62 expression in SH-SY5Y cells treated with or without 20 μM CQ, under the IR model. Autophagic flux of LC3 (panels (**A’**,**B’**)) and p62 (panels (**C’**,**D’**)) represents the difference in protein expression with and without 20 μM CQ. Values are presented as means ± SEM of 4 experiments conducted in duplicate. Red asterisks (*): significant differences in protein expression compared to IR. Green asterisks (*): significant differences in protein expression under different experimental conditions in the absence or presence of CQ (* *p* < 0.05; ** *p* < 0.01; *** *p* < 0.001; one-way ANOVA followed by Holm–Sidak post hoc test).

Conversely, most compounds exhibited a tendency to decrease both LC3 lipidation (especially at 10 μM) (Figure 5B) and p62 expression (primarily at 1 μM) (Figure 5C). These effects were generally reversed by cotreatment with CQ, indicating inhibition of the elongation phase (Figure 5A,B) and enhancement of the degradation phase (Figure 5C,D). **ChN2** was the only compound that did not reduce LC3-II accumulation (Figure 5A,B) and, at 10 μM, displayed a trend towards increased p62 expression, suggesting a potential inhibitory role of the degradation phase (Figure 5D).

To study the effect of these concentrations of the nitrones on the elongation and degradation phases more precisely, we analyzed autophagic flux by calculating the differences in compound effects with and without CQ.

Figure 5A’ shows that all compounds at 1 μM, except **ChN2**, significantly increase autophagic flux. However, at 10 μM, this effect was only statistically significant for **QN6**, while **ChN2** significantly decreased autophagic flux (Figure 5B’).

Regarding p62, autophagic flux significantly increased with all compounds at 1 μM, except for **ChN2**, with the greatest effects observed for **HBN6** and **QN6** (Figure 5C’). Conversely, at 10 μM, there was a downward trend in autophagic flux, significant only for **ChN2** (Figure 5D’).

Overall, these results indicate that five of the tested compounds decrease IR-induced autophagy at low concentrations and have no effect at higher concentrations. On the other hand, **ChN2** shows no apparent effects at 1 μM and increases autophagy at 10 μM.

#### 3.3.2. Effects on Cell Viability in the Absence and Presence of Chloroquine

**Figure 6:**
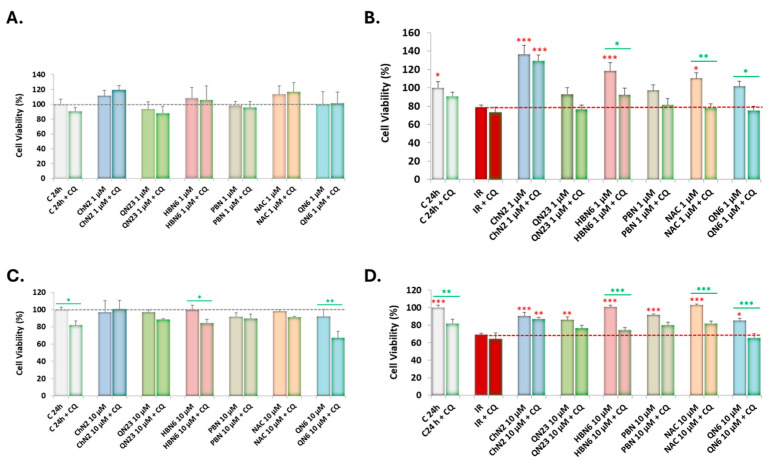
Effects of treatment with study compounds at 1 and 10 μM on the viability of SH-SY5Y cells under basal conditions (**A**,**C**) and IR conditions (**B**,**D**), in the absence or presence of 20 μM CQ. Values shown are the means ± SEMs of three experiments performed in triplicate. Red asterisks (*): significant differences in cellular metabolic capacity compared to the IR condition. Green asterisks (*): significant differences in cell viability under a certain experimental condition and its cotreatment with CQ (* *p* < 0.05; ** *p* < 0.01; *** *p* < 0.001; one-way ANOVA followed by Holm–Sidak post hoc test).

Upon validating the regulatory effects of the compounds on the expression of various autophagic proteins, we investigated whether there was a relationship between this parameter and the neuroprotective potential of the compounds. We evaluated cell viability using the XTT assay after treating SH-SY5Y cells with the six compounds at 1 and 10 μM, in the presence or absence of 20 μM CQ (Figure 6).

Treatment with nitrones at 1 μM and CQ under basal conditions did not affect cellular metabolic capacity (Figure 6A). However, under IR conditions, CQ significantly reduced the neuroprotective effect of all compounds, especially **HBN6**, **NAC**, and **QN6** (Figure 6B). In contrast, the neuroprotective capacity of **ChN2** against ischemic insult was not affected by CQ treatment, indicating that autophagy is not one of the underlying mechanisms for the neuroprotective effect of this nitrone at 1 μM (Figure 6B).

On the other hand, CQ treatment did slightly reduce cell viability under basal conditions in the presence of **HBN6** and **QN6** at 10 μM (Figure 6C), suggesting that autophagy-modulating mechanisms partly account for the neuroprotective effects of these two nitrones in the absence of ischemic insult. Under IR conditions, as expected based on our previous results [34,43,44], all compounds exhibited neuroprotective effects (Figure 6D). However, CQ treatment significantly inhibited the neuroprotective effect of only **HBN6**, **NAC**, and **QN6** (Figure 6D). Consequently, these are the only three compounds that rely on autophagy-modulating mechanisms to exert their neuroprotective role at 10 μM.

### 3.4. Relationship between the Neuroprotective Capacity of the Studied Nitrones and Their Modulatory Effect on Autophagy

#### 3.4.1. Effect of the Studied Nitrones on Autophagy at Different Concentrations: Dose–Response Curves

Given the varying modulatory effects on autophagy observed at different nitrone concentrations, we decided to analyze their impact on the expression of the four selected autophagic markers across a broader concentration range.

**Figure 7:**
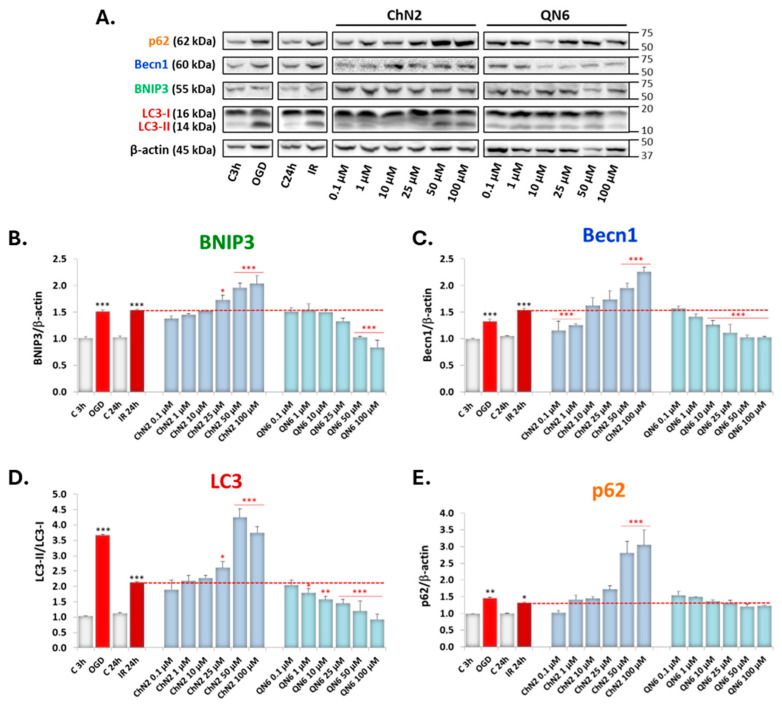
Concentration–response curves showing the effect of nitrones ChN2 and QN6 on the expression of (**A**,**B**) BNIP3, (**A**,**C**) Becn1, (**A**,**D**) LC3, and (**A**,**E**) p62 in human neuroblastoma SH-SY5Y cells exposed to the ischemia–reperfusion experimental model. Values shown are the means ± SEMs of 3 experiments performed in duplicate. Black asterisks (*): significant differences in protein expression under OGD and IR conditions compared to their respective controls (C 3 h and C 24 h, respectively). Red asterisks (*): significant differences in protein expression between different experimental conditions and IR (* *p* < 0.05; ** *p* < 0.01; *** *p* < 0.001; one-way ANOVA followed by Holm–Sidak post hoc test).

We first tested concentrations from 0.1 μM to 100 μM for the two nitrones that showed the most polarizing effects in previous experiments: **QN6**, due to its consistent antiautophagic effects, and **ChN2**, which exhibited an apparent upregulation of certain phases of the autophagic process (Figure 7).

As shown in Figure 7, treatment with **ChN2** increased the expression of the four selected autophagic markers in a concentration-dependent manner, starting at 25 μM for BNIP3 and LC3 (Figure 7B,D) and at 50 μM for Becn1 and p62 (Figure 7C,E). However, at 0.1 and 1 μM, **ChN2** showed a tendency to downregulate the expression of all four proteins, which was statistically significant for Becn1 (Figure 7C).

In contrast, the effect of **QN6** was inhibitory, significantly reducing the lipidation of LC3 starting at 1 μM (Figure 7D) and the expression of Becn1 and BNIP3 starting at 10 and 50 μM, respectively (Figure 7B,C). In these experiments, however, **QN6** did not have significant effects on the expression of p62 (Figure 7E).

**Figure 8:**
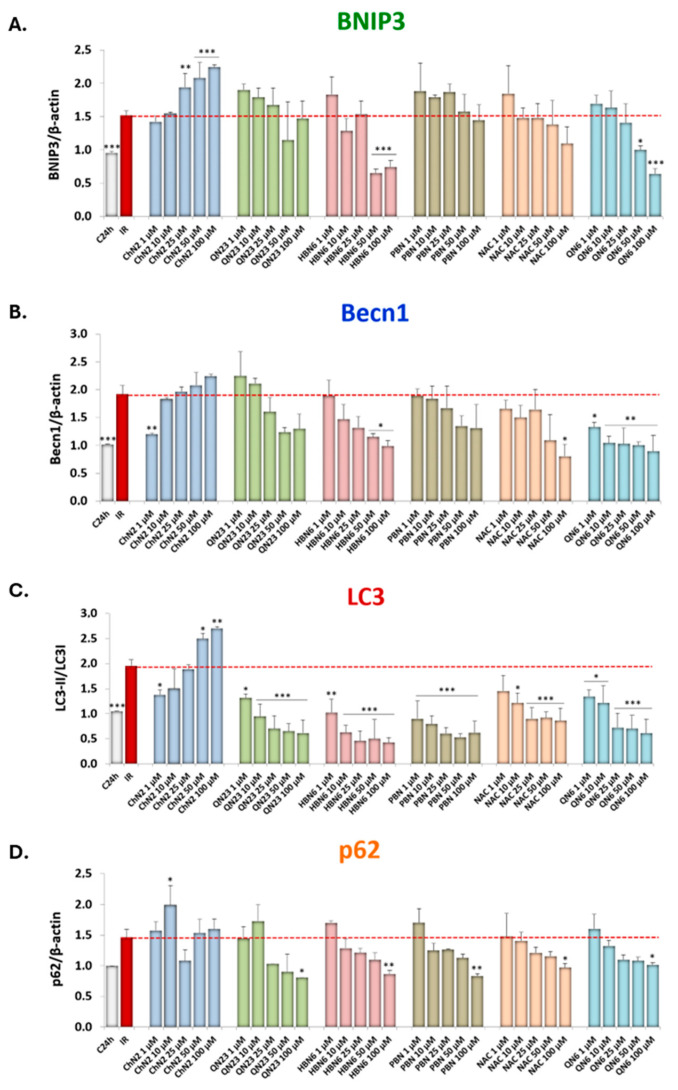
Concentration–response curves showing the effect of the six tested compounds on the expression of (**A**) BNIP3, (**B**) Becn1, (**C**) LC3, and (**D**) p62 in SH-SY5Y human neuroblastoma cells exposed to the ischemia–reperfusion experimental model. Values shown are the means ± SEMs of four experiments performed in duplicate. Asterisks (*): significant differences in protein expression compared to IR in the absence of compounds (* *p* < 0.05; ** *p* < 0.01; *** *p* < 0.001; one-way ANOVA followed by Holm–Sidak post hoc test).

To increase the sample size and extend the study to all five nitrones and **NAC**, experiments were conducted with all compounds over a concentration range of 1 to 100 μM (Figure 8).

For BNIP3 (Figure 8A), notable modulatory effects were observed only at the highest concentrations of some compounds. **ChN2** exhibited a promoting effect on induction and nucleation, beginning at 25 μM, while **HBN6** and **QN6** demonstrated inhibitory effects, starting at 50 μM.

In contrast, the compounds predominantly showed antiautophagic properties in regulating Becn1 expression. **ChN2** significantly reduced its expression at low concentrations (1 μM), while **HBN6** (at 50 and 100 μM) and **NAC** (at 100 μM) exerted similar effects at higher concentrations (Figure 8B). **QN6** consistently reduced Becn1 expression across all tested concentrations (Figure 8B).

All compounds, except **ChN2**, significantly reduced LC3 lipidation across nearly all concentrations (except for **NAC** at 1 μM), thereby downregulating the autophagic elongation phase (Figure 8C). **ChN2** decreased this parameter at the lowest concentration of 1 μM but significantly increased it at 50 and 100 μM (Figure 8C).

Lastly, at 10 μM, **ChN2** demonstrated an inhibitory effect on the degradation phase, increasing p62 expression levels. The other five compounds exhibited the opposite effect, albeit statistically significant only at 100 μM (Figure 8D).

All these results clearly indicate that, with the exception of **ChN2**, which promotes autophagy at high concentrations, the tested compounds inhibit autophagy in a concentration-dependent manner, following this order based on the potency of their antiautophagic effects: **QN6** ≥ **HBN6** > **NAC** ≥ **QN23** ≥ **PBN**.

#### 3.4.2. Linear Correlation Between Anti- or Proautophagic Effects and the Neuroprotective Capacity of the Tested Nitrones

Given that the neuroprotective mechanisms of the studied compounds also involve regulation of other types of cell death (primarily necrosis and apoptosis) [34,43,44], we aimed to elucidate the role of autophagy modulation in their overall neuroprotective efficacy. For this, we performed linear correlation analyses between the % of autophagy regulatory effects (considering variations in BNIP3, Becn1, LC3, and p62 protein expression) and the % of overall neuroprotective effect at the different tested concentrations (1–100 μM) for each compound.

**Figure 9:**
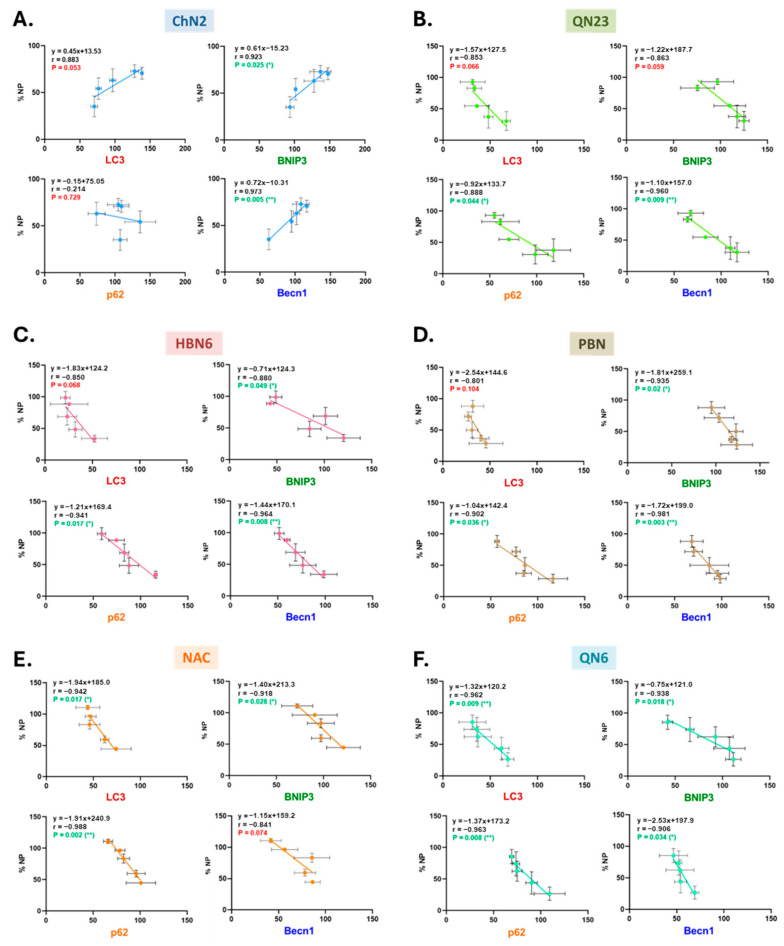
Correlation analyses between autophagic modulation and the overall neuroprotection exerted by the tested compounds. Linear regression analyses show the correlation between the expression (%) of different autophagic markers (LC3, p62, Becn1, and BNIP3) and overall neuroprotection (NP) (%) in the presence of (**A**) **ChN2**, (**B**) **QN23**, (**C**) **HBN6**, (**D**) **PBN**, (**E**) **NAC**, and (**F**) **QN6**. Straight line equations (ax + b), correlation coefficients (r), and the statistical significance of the regression analyses are indicated in each plot. Regression analyses and statistics were performed using the Pearson correlation test at * *p* < 0.05 and ** *p* < 0.01.

As shown in Figure 9, except for **ChN2** (Figure 9A), all correlations were negative, indicating that the reduction in autophagy induced by the tested nitrones correlates with an increase in their overall neuroprotective capacity. Nevertheless, there were differences among the nitrones regarding the statistical significance of the correlation between their neuroprotective effects and the regulation of the four autophagic markers’ expression.

For quinolylnitrone **QN6** (Figure 9F), all correlations were statistically significant, suggesting that its neuroprotective effect heavily depends on its ability to inhibit the induction, nucleation, elongation, and degradation phases of autophagy.

With **NAC** (Figure 9E), the correlation between BNIP3 expression and the overall neuroprotective effect was not statistically significant, but the correlation between Becn1 expression and the overall neuroprotective effect was. This indicates that **NAC**’s antiautophagic effect primarily occurs through the inhibition of autophagic flux during the elongation and degradation phases.

Regarding **HBN6** (Figure 9C) and **PBN** (Figure 9D), the correlation between LC3 expression and their overall neuroprotective effect was not significant, suggesting that the antiautophagic effects of these two nitrones mainly depend on the inhibition of the initiation, nucleation, and degradation phases of autophagy.

In the case of **QN23** (Figure 9B), the correlations related to BNIP3 and LC3 were not statistically significant, implying that its antiautophagic effect primarily occurs during the final stages of autophagy.

Lastly, **ChN2** (Figure 9A) stands out as its overall neuroprotective effect appeared to depend on its capacity to promote autophagy, unlike the rest of the compounds. However, the nonsignificant correlations for LC3 and p62 expression in **ChN2**’s case suggest a milder effect on autophagy, and that its slight upregulatory effect on the initial stages might aid in inhibiting apoptosis and necrosis. At higher concentrations, its neuroprotective effect no longer relies on autophagy activation but, rather, on its antiapoptotic and antinecrotic properties, as observed in our previous studies [44], where this nitrone was one of the most potent.

## 4. Discussion

This study aimed to deepen our understanding of the role of autophagy in the pathophysiology of stroke by analyzing the regulation of molecular markers characteristic of different phases of the autophagic process under the conditions of an in vitro ischemia–reperfusion (IR) model. Additionally, we examined the effects of treatment with four synthetic nitrones, selected for their demonstrated in vitro antinecrotic, antiapoptotic, and antioxidant potential [34,38,39,41,43,44], as well as their in vivo neuroprotective effect, in cerebral ischemia models shown in previous studies [34,39,41,42,45].

In order to achieve this, we employed cultures of the human neuroblastoma cell line SH-SY5Y, a well-established model widely used in neuroprotection studies related to ischemic stroke [59,60]. Cell line experiments have limitations, as they do not fully replicate the physiological conditions of a biological system, but they also offer several benefits, as they are accessible, easy to maintain, provide an abundant supply of cells, and allow precise control over environmental variables, thus enhancing reproducibility [59,61]. Moreover, human in vitro systems are frequently used for cost-effective high-throughput screening assays to evaluate the efficacy of therapeutic compounds [59,61].

### 4.1. The Ischemia–Reperfusion Model Promotes Prodeath Autophagy by Enhancing the Induction, Nucleation, and Elongation Phases of This Process

To investigate the influence of autophagy on the effects of the IR model, BNIP3 and Becn1 were chosen as molecular markers for the initiation and nucleation phases [11,20], LC3 was selected as a marker for the elongation phase due to its lipidation and incorporation into autophagosome membranes [6,12], and p62 was used as a marker of the degradation phase, since its levels are directly related to the autolysosomal degradation rate [6,27].

Firstly, our results showed an increase in the expression of the two induction/nucleation phase markers under ischemia–reperfusion model conditions (Figure 2A,B). Under oxygen–glucose deprivation, there was a significant increase in LC3 lipidation, which was partially reversed following reperfusion (Figure 2C). Furthermore, under OGD and IR conditions, and with rapamycin treatment (a positive control for autophagy) [1,51], we observed a marked increase in autophagic flux (expressed as an increase in LC3-II and p62 accumulation under these experimental conditions, in cotreatment with CQ) (Figure 2C’,D’). Consequently, the IR model used appears to promote autophagy by enhancing the induction, nucleation, and elongation phases.

It is important to note that calculating autophagic flux based on changes in the expression of autophagy molecular markers in the absence and presence of CQ serves only as an indicator of this parameter. This method helps to create a “snapshot” of the accumulation of these proteins by blocking autophagic flux through lysosomal pH acidification [49] and inhibiting autophagosome–lysosome fusion [50]. However, since flux is a rate, a proper measurement of autophagic flux must necessarily consider the temporal factor through techniques that facilitate a dynamic study of this parameter [62]. Therefore, to confirm our results on autophagic flux and validate the conclusions, an evaluation of lysosomal function would be required [49].

Subsequently, we observed a decrease in cell viability under basal conditions with CQ treatment (Figure 3), which may indicate the presence of prosurvival autophagy mechanisms underlying the maintenance of the metabolic capacity of SH-SY5Y cells in the absence of damage conditions. Under IR conditions, CQ had no significant effects on cell viability, but treatment with Rapa reversed the decrease in cell survival caused by exposure to the IR model (Figure 3). It is important to emphasize that rapamycin is a “pure” compound that acts solely by promoting autophagy through the inhibition of mTOR phosphorylation [6,51,63], thereby regulating key cellular functions such as protein translation, cell growth, and metabolism [64,65]. In contrast, chloroquine targets multiple molecular pathways [50,66,67]. Additionally, the decrease in cell survival due to IR exposure depends on various cell death mechanisms (necrosis, apoptosis, pyroptosis, ferroptosis) [8,10]. Consequently, the effects of CQ on cell viability under ischemia–reperfusion conditions may not be solely due to autophagy regulation. However, we can conclude that rapamycin appears to enhance beneficial prosurvival autophagy mechanisms in the presence of ischemic insult, while it does not affect cell viability under basal conditions (Figure 3). These results are consistent with those of other authors who have demonstrated that rapamycin offers neuroprotection in in vitro models of Alzheimer’s (AD), Huntington’s (HD), and Parkinson’s (PD) disease and in in vivo models of AD, reducing β-amyloid and tau accumulation and improving cognitive function [68].

### 4.2. HIF-1α Acts as a Primary Inducer of Autophagy during Ischemia and the Early Stages of Reperfusion

After confirming the involvement of autophagy in the underlying mechanisms of the IR model, we investigated potential inducers of this process. We hypothesized that HIF-1α could play a role, given its critical function in cellular responses to hypoxia [16,53]. Our results demonstrated a significant increase in HIF-1α expression under OGD conditions, which progressively reversed as reperfusion time increased (Figure 4B). Quantitative analysis indicated that HIF-1α protein levels increased significantly in the presence of PHD inhibitors—which prevent HIF-1α degradation—only during short reperfusion periods (Figure 4B). Furthermore, PHDIs elevated LC3 lipidation at 3 h of reperfusion, but both LC3 lipidation and BNIP3 expression decreased at 24 h of reperfusion (Figure 4C,D). These findings suggest that autophagy induced by IR is initially triggered by HIF-1α. However, at extended reperfusion times, autophagy may become independent of HIF-1α, possibly sustained by other mechanisms such as AMPK activation and inhibition of mTOR phosphorylation [13,58].

### 4.3. The Neuroprotective Effects of Most Examined Compounds Partly Derive from Their Suppression of Autophagic Cell Death

Next, we analyzed whether the four synthetic nitrones selected in this study had modulatory effects on IR-induced autophagy, comparing them with two reference compounds: α-phenyl-N-tert-butylnitrone (**PBN**) and N-acetylcysteine (**NAC**). In an initial approach using only two concentrations of the compounds (1 and 10 μM), we observed: (1) a trend towards downregulating the induction and nucleation phases (particularly by **QN6**, which significantly reduced Becn1 expression at 1 μM and BNIP3 expression at 10 μM) (Figure S2); and (2) a general tendency to decrease LC3 lipidation (especially at 10 μM) and p62 expression (mainly at 1 μM) (Figure 5). The only nitrone that did not follow this pattern was **ChN2**, which did not alter LC3 lipidation (Figure 5A,B) and appeared to increase p62 expression at 10 μM (Figure 5D).

When we evaluated autophagic flux variations considering the effects of combined treatment with CQ, we observed a tendency for all compounds—except **ChN2**—to increase this parameter at 1 μM (Figure 5A’,C’). However, at 10 μM, only **QN6** maintained this trend, while **HBN6**, **PBN**, and **NAC** ceased to show significant effects on flux, and **QN23** tended to decrease it (Figure 5B’,D’). Thus, these five compounds seem to increase beneficial autophagic flux and degradation rates at low concentrations but inhibit the elongation phase at higher concentrations. Future studies are needed to examine the effects of concentrations higher than 10 μM to confirm this hypothesis. Meanwhile, **ChN2** did not show significant effects at 1 μM but significantly reduced autophagic flux at 10 μM (Figure 5B’,D’). It is crucial to distinguish between monitoring steady-state levels of LC3 and autophagic flux. Although we evaluated the latter by studying LC3 and p62 expression in the absence and presence of CQ, we did not assess autophagy-dependent degradation of appropriate substrates. Therefore, to confirm our results and validate conclusions about autophagic flux, an evaluation of lysosomal function is required [49].

When we assessed the effects of these experimental conditions on cell viability, we found that cotreatment with CQ significantly reduced the increase in cellular metabolic capacity induced by **QN23**, **HBN6**, **PBN**, **NAC**, and **QN6** at 1 μM (Figure 6B), and by **HBN6**, **NAC**, and **QN6** at 10 μM (Figure 6D). This indicates that autophagy-modulating mechanisms may underlie their neuroprotective effects against IR at these concentrations. Under basal conditions, combined treatment with CQ only significantly decreased viability with **HBN6** and **QN6** at 10 μM (Figure 6C), suggesting that autophagy regulation could be implicated in their impact on cellular metabolic capacity in the absence of ischemic insult.

Expanding the concentration range studied (1–100 μM), all compounds except **ChN2** exhibited clear antiautophagic effects (Figure 7 and Figure 8). **HBN6,** and particularly **QN6,** demonstrated the most significant effects, inhibiting the induction and nucleation phases at medium–high concentrations, significantly downregulating the elongation phase across nearly all tested concentrations, and enhancing the degradation phase only at 100 μM (Figure 8). **QN23**, **PBN**, and **NAC** displayed similar impacts on the elongation and degradation phases but did not significantly influence induction/nucleation marker expression, except for **NAC**, which reduced Becn1 expression at 100 μM (Figure 8B). Conversely, **ChN2** exhibited distinct proautophagic effects at medium–high concentrations, promoting induction, nucleation, and elongation from 25 μM and tending to inhibit degradation (Figure 7 and Figure 8). Notably, at the lowest concentrations, **ChN2** followed a similar trend to the other compounds, reducing the expression of all molecular markers (Figure 7 and Figure 8).

### 4.4. The Neuroprotective Effects of Nitrones in Ischemia–Reperfusion Correlate with Their Inhibition of Various Autophagic Phases

Finally, we aimed to determine if there was a relationship between the autophagy-modulating effects of the compounds and their overall neuroprotective capacity, previously demonstrated in earlier studies by our group [34,43,44]. Analyses for all compounds except **ChN2** revealed a negative correlation between these parameters (Figure 9), indicating that their antiautophagic effects were, to some extent, responsible for their neuroprotective power. For **QN6**, the correlations between the decreased expression of the four autophagy molecular markers and its neuroprotective effects from 1 to 100 μM were all statistically significant (Figure 9F). Therefore, it appears that **QN6**’s neuroprotective properties are strongly dependent on its inhibitory effects on the induction, nucleation, and elongation phases and its tendency to enhance the degradation phase. The results for the other four compounds suggest that their neuroprotective effects depend on modulating specific phases of autophagy, but not all of them entirely. **NAC**’s effect is not reliant on induction/nucleation inhibition, **HBN6** and **PBN**’s effects do not depend on the downregulation of elongation, and **QN23**’s neuroprotective effect appears to primarily depend on its promotion of the degradation phase (Figure 9B–E). Lastly, **ChN2** stands out as its overall neuroprotective capacity seems to rely on its promotion of the early phases of autophagy, potentially contributing to the inhibition of necrosis and apoptosis, while the correlations with the increase it causes in LC3 lipidation and p62 expression are not statistically significant (Figure 9A).

Although the results of this work are limited to in vitro experiments carried out in cultured neuronal cells, they underscore the critical role of autophagy modulation in the neuroprotective effects of compounds with proven efficacy in preclinical models of cerebral ischemia. These findings highlight the importance of promoting prosurvival autophagy and, specially, inhibiting prodeath autophagy as promising therapeutic approaches in treating ischemic stroke clinically.

## 5. Conclusions

**Figure 10:**
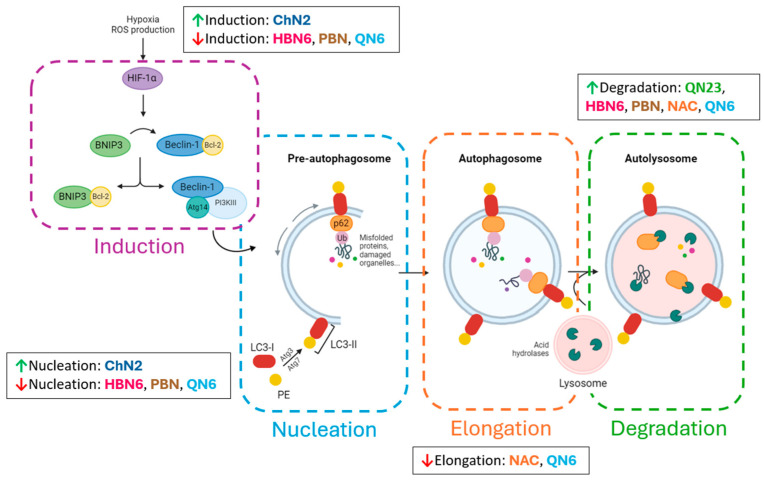
Modulatory effects of the study compounds on the phases of autophagy. **HBN6** and **QN6** have the most significant effects, inhibiting the induction and nucleation phases at medium–high concentrations, downregulating the elongation phase across most concentrations, and enhancing the degradation phase only at 100 μM. **QN23**, **PBN**, and **NAC** similarly affect elongation and degradation, but do not significantly influence induction or nucleation. In contrast, **ChN2** exhibits distinct proautophagic effects at medium–high concentrations, promoting induction and nucleation and tending to inhibit degradation.

In conclusion, this study elucidates several key findings (Figure 10):

(1) Autophagy plays a crucial role in cell death induced by an in vitro ischemia–reperfusion (IR) model using a human neuroblastoma cell line. IR impacts multiple phases of autophagy—from initiation and nucleation to elongation and lysosomal degradation—manifested through alterations in specific protein markers (BNIP3, Becn1, LC3, and p62).
(2) HIF-1α acts as a primary inducer of early autophagy during ischemia, potentially complemented by inhibition of the mTOR pathway in subsequent stages.
(3) The neuroprotective effects of most examined nitrones (excluding **ChN2**) and **NAC** stem partially from their suppression of autophagic cell death, marked by significant reductions in proteins involved across various autophagic phases. Conversely, **ChN2**’s neuroprotection primarily results from its antinecrotic and antiapoptotic properties; its antiautophagic effects are minimal at low concentrations, but high concentrations elicit prodeath autophagy, particularly in initial phases.

These findings suggest that inhibiting, rather than stimulating, autophagy may offer a promising therapeutic approach for ischemic stroke, in contrast to strategies employed for other neurodegenerative conditions.

## Figures and Tables

**Table 1:** Antibodies (Ab) used for Western blot.

Primary Ab	Concentration	Producer	Molecular Weight (kDa)	Secondary Ab	Concentration	Producer
Rabbit anti- LC3A/B	1:1000	Cell Signaling (Madrid, Spain)	14, 16	HRP-conjugated goat anti-rabbit	1:5000	Invitrogen (Madrid, Spain)
Mouse anti- BNIP3	1:1000	Abcam (Madrid, Spain)	22–28, 50–55	HRP-conjugated goat anti-mouse	1:4000	Sigma-Aldrich (Madrid, Spain)
Mouse anti- β-actin	1:6000	Sigma-Aldrich (Madrid, Spain)	45	HRP-conjugated goat anti-mouse	1:4000	Sigma-Aldrich (Madrid, Spain)
Rabbit anti- Beclin-1	1:1000	Cell Signaling (Madrid, Spain)	60	HRP-conjugated goat anti-rabbit	1:5000	Invitrogen (Madrid, Spain)
Rabbit anti- p62	1:500	Cell Signaling (Madrid, Spain)	62	HRP-conjugated goat anti-rabbit	1:5000	Invitrogen (Madrid, Spain)
Mouse anti- HIF-1α	1:1000	R&D Systems (Madrid, Spain)	120	HRP-conjugated goat anti-mouse	1:4000	Sigma-Aldrich (Madrid, Spain)

## Data Availability

The original contributions presented in the study are included in the article/Supplementary Materials, further inquiries can be directed to the corresponding author.

## Supplementary Materials

The following supporting information can be downloaded at: https://www.mdpi.com/article/10.3390/antiox13080946/s1, Figure S1: Molecular targets for the modulation of different stages of autophagy. Figure S2: Effect of study compounds at 1 and 10 μM on the expression of (A,B) BNIP3 and (C,D) Becn1 in SH-SY5Y cells treated with or without 20 μM CQ, under the conditions of the experimental IR model.
