# Supplementary material for: In Vitro Modulation of Autophagy by New Antioxidant Nitrones as a Potential Therapeutic Approach for the Treatment of Ischemic Stroke
Source: Antioxidants (Basel). 2024 Aug 3;13(8):946. doi: 10.3390/antiox13080946 (PMC11351736; doi:10.3390/antiox13080946)
Supplement: Supplementary file 1 [file antioxidants-13-00946-s001.zip › antioxidants-3108765-supplementary.pdf]

Supplementary Material

In Vitro Modulation of Autophagy by New Antioxidant Nitrones as a Potential Therapeutic Approach for the Treatment of Ischemic Stroke

**Sara Izquierdo-Bermejo [1,2,3], Beatriz Chamorro [1,3], María Dolores Martín-de-Saavedra [1,2,4], Miguel Lobete [1,2],
Francisco López-Muñoz [3,5,6], José Marco-Contelles [7,8] and María Jesús Oset-Gasque [1,2,4,*]**

1 Department of Biochemistry and Molecular Biology, Faculty of Pharmacy, Complutense University of Madrid, Plaza Ramón y Cajal s/n, Ciudad Universitaria, 28040 Madrid, Spain; sizqui03@ucm.es (S.I.-B.); beatrcha@ucm.es (B.C.); marmar68@ucm.es (M.D.M.-d.-S.); milobete@ucm.es (M.L.)
2 Instituto de Investigación Sanitaria del Hospital Clínico San Carlos, 28040 Madrid, Spain
3 Faculty of Health Sciences–HM Hospitals, Camilo José Cela University, Villafranca del Castillo, 28692 Madrid, Spain; flopez@ucjc.edu
4 Instituto Universitario de Investigación en Neuroquímica, Complutense University of Madrid, Ciudad Universitaria, 28040 Madrid, Spain
5 HM Hospitals Health Research Institute, 28015 Madrid, Spain
6 Neuropsychopharmacology Unit, "Hospital 12 de Octubre" Research Institute, 28041 Madrid, Spain
7 Laboratory of Medicinal Chemistry, Institute of Organic Chemistry (CSIC), C/Juan de la Cierva 3, 28006 Madrid, Spain; jlmarco@iqog.csic.es
8 Center for Biomedical Network Research on Rare Diseases (CIBERER), Carlos III Health Institute (ISCIII), 28029 Madrid, Spain
* Correspondence: mjoset@ucm.es; Tel.: +34-1-394-1788

Content

Figure S1……………………………………………………………………………………………….. 2S
Figure S1……………………………………………………………………………………………….. 3S

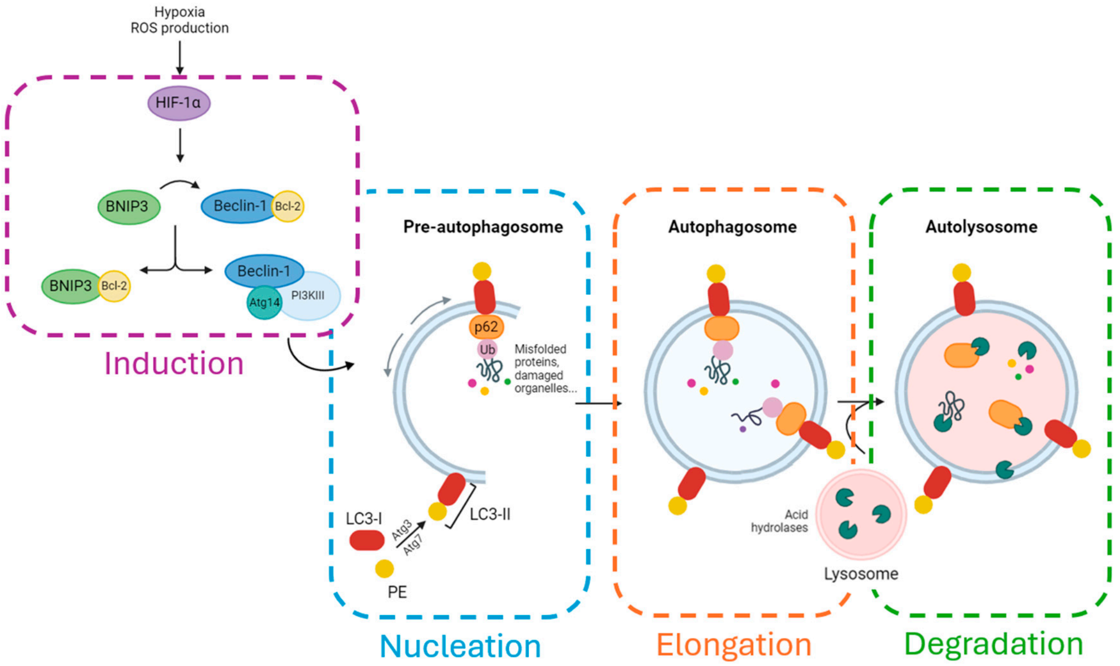

Figure S1. Molecular targets for the modulation of different stages of autophagy. Hypoxia and ROS production can trigger the induction of autophagy through HIF-1*α* [17,18]. During the initiation phase, this transcription factor induces the expression of BNIP3, which binds to Bcl-2, enabling the interaction between Becn1 and PI3K-III [20], and triggering nucleation. Then, in the elongation phase, LC3-I is lipidated by the addition of a phosphatidylethanolamine (PE) molecule [6,11]. The lipidated form of LC3, known as LC3-II, is incorporated into the autophagosome membrane and binds to autophagic adapters such as p62, facilitating substrate identification for degradation [9,27]. During this process, both adapters and non-functional cellular proteins and organelles are eliminated by lysosomal hydrolases (i.e., cathepsines, acid phosphatases) inside the autolysosome.

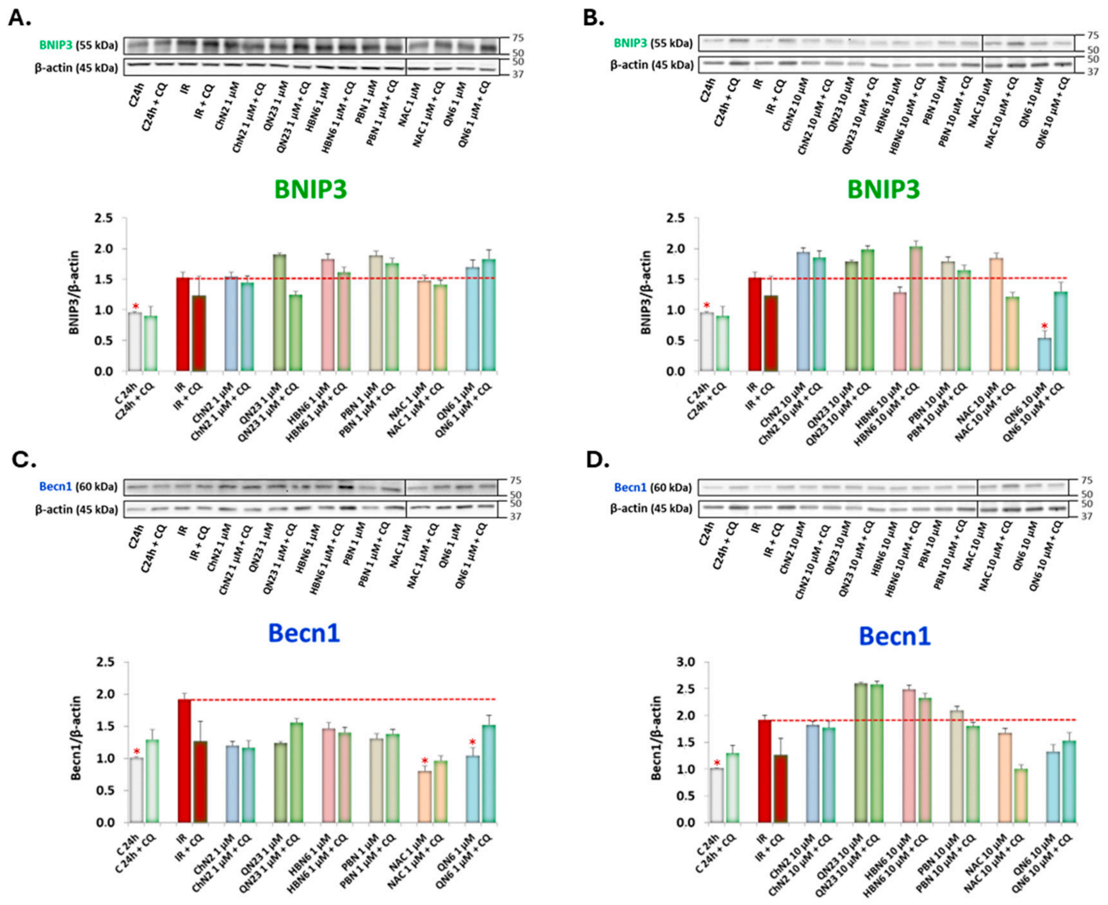

Figure S2. Effect of study compounds at 1 and 10 µM on the expression of (A, B) BNIP3 and (C, D) Becn1 in SH-SY5Y cells treated with or without 20 µM CQ, under the conditions of the experimental IR model. Values are presented as means ± SEM of 4 experiments conducted in duplicate. Red asterisks (*): significant differences in protein expression compared to IR (* $p < 0.05$; one-way ANOVA followed by Holm–Sidak post hoc test).
